# Survival and relapse of Danish patients with thymic epithelial tumors

**DOI:** 10.2340/1651-226X.2026.45407

**Published:** 2026-05-21

**Authors:** Tine Østergaard, Caroline Van Alstine Bjerke, Eric Santoni-Rugiu, Thomas Hartvig Lindkær Jensen, Katharina Anne Perell, René Horsleben Petersen, Peter Meidahl Petersen

**Affiliations:** a Department of Oncology, Copenhagen University Hospital, Copenhagen, Denmark; b Department of Pathology, Copenhagen University Hospital, Copenhagen, Denmark; c Department of Cardiothoracic Surgery, Copenhagen University Hospital, Copenhagen, Denmark; d Department of Clinical Medicine, University of Copenhagen, Copenhagen, Denmark

**Keywords:** Thymic epithelial tumor, thymoma, thymic carcinoma, progression-free survival, prognosis, recurrence, mortality

## Abstract

**Background and purpose:**

This study aims to evaluate survival, relapse, comorbidities, and prognostic factors in patients with thymic epithelial tumours (TETs) to improve risk stratification for TET-related mortality and relapse and support evidence-based follow-up and treatment strategies.

**Patient/material and methods:**

The cohort consists of consecutive Danish patients diagnosed with thymoma, thymic carcinoma (TC), or thymic neuroendocrine tumor (NET) between January 1st, 2015 and December 31st, 2020, with follow-up data available.

Data were collected from online medical records including disease characteristics, survival outcomes, comorbidities, and relapse. Statistical analyses were performed in R 4.3.2 and the prognostic value baseline traits were analyzed using Cox Proportional Hazards models.

**Results:**

Our cohort comprised 282 patients with a mean diagnostic age of 65 years and the following histological distribution: thymoma (85%), TC (13.6%), and NET (1.4%). During the 72-month mean follow-up, 65 deaths occurred yielding overall survival (OS) of 81% in patients with thymoma and 47% in patients with TC. TET-related deaths predominated in TC, yielding a cancer-specific survival) of 53% compared with 96% in thymoma. Reduced OS was associated with increasing age (*p* < 0.001) and advanced Tumor, Node, Metastasis-stage (III *p* < 0.05; IV *p* < 0.001). Disease progression was verified in 13/201 (6%) of thymoma patients with R0 or R1 resection.

**Interpretation:**

We report a high OS of Danish TET patients compared to existing population-based studies. The occurrence of TET-related mortality and disease progression in patients with thymoma and TC, highlights the need for long-term follow-up and therefore risk stratification in TET management.

## Introduction

Tumors derived from the thymic epithelium are the most common malignancies of the anterior mediastinum, although they are very rare and occur in 0.48–0.5 per 100,000 [1, 2]. Thymic epithelial tumors (TETs) are divided in three histopathological distinct types, of which thymomas are the most frequent, thymic carcinomas (TC) the most malignant, and thymic neuroendocrine tumors (NET) the rarest [3].

This diverse nature of the different types of TETs is reflected in the survival outcomes, with the 5-year cancer-specific survival (CSS) ranging from 91% for thymoma to 50% for TC [1]. Patients with TETs are found to be at risk of relapse even several years after conclusion of primary treatment [4, 5].

Autoimmune diseases and other primary malignancies are more prevalent in patients with TETs, with the association most pronounced in patients with thymoma [6, 7]. Myasthenia Gravis (MG) is the most frequent autoimunne disease and is diagnosed in 15–35% of TET patients [4, 7, 8]. Historical reports find autoimmune diseases and MG to be the cause of death in, respectively, 7 and 3% of patients with thymoma [9, 10]. Furthermore, TET patients have a two-fold increased risk of developing other primary cancers compared to the general population [6, 11, 12] and have correspondingly higher proportion of deaths caused by other cancers (2.4–8.5%) compared to patients with other primary cancers [1].

A multidisciplinary approach is essential for effective management of TETs due to their clinical heterogeneity and associated risk of relapse and paraneoplastic complications.

In line with European Society for Medical Oncology (ESMO) guidelines, Danish protocols recommend complete resection as primary objective for patients with localized thymomas and TC and chemo- or radiotherapy for unresectable tumors [13, 14] with a similar approach at relapse.

After the conclusion of primary treatment, all TET patients are enrolled in a formalized follow-up programs comprising regular computer tomography (CT)-scans, blood samples and clinical assessments to enable timely discovery of disease relapse or development of TET-associated autoimmune or malignant comorbidities. At present, the follow-up program is designed according to a simple risk evaluation of the patient including tumor histology, stage and resection status [13, 14]. This is based on limited evidence, not accurately reflecting the current composition and risk of the Danish patients.

This study aims to evaluate survival outcomes, relapse rates, comorbidity risks, and prognostic factors in a comprehensive national cohort of Danish TET patients to enhance risk stratification for TET-related mortality and relapse. This enables more evidence-based follow-up and treatment strategies for TET patients.

## Patients/material and methods

To ensure the best possible quality standards, care of all Danish TET patients is centralized in one national centre. The diagnostics and treatment of TET represents a multidisciplinary effort across oncologists, pathologists, radiologists, neurologists, and thoracic surgeons [14].

Since its establishment in 2021, the Danish TET Database has collected comprehensive data on disease, treatment, and complications of Danish TET patients to facilitate research and inform evidence-based patient care [2].

### Patients

Using the Danish Pathology Data Bank (DPDB) we identified 282 patients with diagnosis of thymoma, TC, or NET from January 2015 to December 2020. The DPDB enables data extraction from the Danish National Pathology Registry (DNPR), which contains all Danish pathology reports since 1997 [15], thus enabling complete national coverage.

### Data collection

Baseline data were collected from online medical records at the time of TET diagnosis and included patient- and disease characteristics and existing comorbidities. Data from patient follow-ups, comprising vital status, cause of death, progression status and development of paraneoplastic comorbidities, was collected from medical records between August 1st and August 31^st^, 2024, and was available for the 282 patients comprising the final study cohort. One patient was censored at follow-up, as follow-up data were unavailable due to emigration.

All histopathologically verified cancers, detected in the medical records were recorded as extrathymic primary cancers. Autoimmune diseases were identified from the medical records based on established diagnoses with a known or strongly suspected etiology.

### Methods

All TETs were histopathologically classified according to the IARC WHO (2021) Guidelines [3]. In concordance with these official guidelines, mixed thymomas and other tumors of mixed histology were classified according to the combined components and the representation of each dominant or minor component with 10% intervals in the tumor tissue [2, 3]. Yet, combined TETs containing both TC and thymoma were clinically considered as TCs due to the more aggressive nature and negative prognostic impact of TC [3]. Mixed-subtype thymomas were categorized according to the volume contribution of individual subtypes, recorded at 10% intervals (Supplementary Table 1) [3].

The 8th edition of the Union for International Cancer Control (UICC) Tumor, Node, Metastasis (TNM) system was used for tumor staging [16]. Tumors initially staged by the Masaoka-Koga system were restaged based on histopathological and radiological assessment reports [3]. Resected tumors were assessed histopathologically and staged according to tumor invasiveness and the radicality of the resection, while clinico-radiological staging was used for patients with no surgical intervention.

Overall survival (OS) was measured as survival from the time of TET diagnosis to death from any cause or last follow-up. Five-year OS was assessed as survival 5 years post diagnosis including only patients followed for at least 5 years or with death occurring within that period, censoring patients followed for less than 5 years.

CSS was assessed as survival from diagnosis until death caused by TET, censoring patients with death from competing causes at the time of death. Similarly, 5-year CSS was calculated as survival 5 years after TET diagnosis considering only patients with follow-up time exceeding or with death occurring within 5 years of diagnosis.

TET relapse was considered as relapse of disease after complete remission, defined as R0 resection. Progression-free survival (PFS) was measured as the time from tumor resection to death due to TET or radiographically verified TET progression for patients with R0 or R1 resections. Imaging data on disease progression was not collected for patients with macroscopically incomplete resections (R2) or for patients with no surgical intervention.

### Data analysis

Statistical analyses were carried out using R 4.3.2 [17] and RStudio 2024.04.2 [18]. The OS of the Danish TET patients was illustrated using the Kaplan–Meier method utilizing the survival (version 3.5.7) and Survminer R packages. Multivariate analyses were conducted using the Cox Proportional Hazards (Cox PH) model and R survival package.

## Results

Between 2015 and 2020, 282 cases of TETs were diagnosed in Denmark. Thymomas comprised 240 (85%) of the 282 TETs, while TCs and NETs accounted for 39 (14%) and 4 (1.4%) of tumors, respectively (Table 1) [2]. As previously reported [2], the predominant subtype was conventional squamous among TC patients, and atypical carcinoid among NET patients.

**Table 1:** Baseline Characteristics of Danish TET Patients

Variable	Thymoma (*N* = 240)	TC (*N* = 38)	NET (*N* = 4)	Overall (*N* = 282)
**Sex**				
Female	128 (53.3%)	15 (39.5%)	0 (0%)	143 (50.7%)
Male	112 (46.7%)	23 (60.5%)	4 (100%)	139 (49.3%)
**Age**	64.6 (± 12.1)	65.3 (± 11.5)	64.8 (± 10.4)	64.7 (± 12.0)
**Tumor histology**				
A	22 (9.2%)			22 (7.8%)
AB	76 (31.7%)			76 (27.0%)
B1	44 (18.3%)			44 (15.6%)
B2	61 (25.4%)			61 (21.6%)
B3	14 (5.8%)			14 (5.0%)
Metaplastic	1 (0.4%)			1 (0.4%)
Micronodular	12 (5.0%)			12 (4.3%)
Squamous		35 (92.1%)		35 (12.4%)
Micronodular TC		2 (5.3%)		2 (0.7%)
NOS TC*		1 (2.6%)		1 (0.4%)
Atypical carcinoid			3 (75.0%)	3 (1.1%)
Large cell NET			1 (25.0%)	1 (0.4%)
**TNM-stage**				
I	172 (71.7%)	6 (15.8%)	1 (25.0%)	179 (63.5%)
II	27 (11.3%)	5 (13.2%)	0 (0%)	32 (11.3%)
III	19 (7.9%)	7 (18.4%)	0 (0%)	26 (9.2%)
IV	22 (9.2%)	20 (52.6%)	3 (75.0%)	45 (16.0%)
**Resection**				
Not resected	21 (8.8%)	18 (47.4%)	0 (0%)	39 (13.8%)
R0	172 (71.7%)	13 (34.2%)	3 (75.0%)	188 (66.7%)
R1	29 (12.1%)	5 (13.2%)	1 (25.0%)	35 (12.4%)
R2	18 (7.5%)	2 (5.3%)	0 (0%)	20 (7.1%)
Existing cancer**				
No	147 (61.3%)	28 (73.7%)	2 (50.0%)	177 (62.8%)
Yes	93 (38.8%)	10 (26.3%)	2 (50.0%)	105 (37.2%)
**Existing autoimmunity**				
No	156 (65.0%)	34 (89.5%)	4 (100%)	194 (68.8%)
Yes	84 (35.0%)	4 (10.5%)	0 (0%)	88 (31.2%)

The categories ‘Existing cancer’ and ‘Existing autoimmunity’ represent the number of patients with established cancer diagnoses and autoimmune diseases prior to TET diagnosis.

* Not otherwise specified (NOS) thymoma and TC includes patients without subtype classification due to insufficient tissue from biopsies.

** When non melanoma skin cancer was excluded, 82 patients (29.1%) had a documented cancer diagnosis preceding TET. TET: thymic epithelial tumours; TC: thymic carcinoma; NET: neuroendocrine tumor; TNM: Tumor, node, metastasis.

Among surgically treated patients, complete (R0) tumor resection was obtained in 172 of 219 (78.5%) patients with thymoma, 13 of 20 (65.0%) with TC, and 3 of 4 (75%) with NET (Supplementary Table 2).

The mean time from TET diagnosis to follow-up was 72 months (range: 44–115 months). A total of 217 patients were followed for more than 5 years after TET diagnosis or died within that time span.

## Cause of death

During the follow-up period, 65 patients (23%) died, with 53 deaths occurring within the first 5 years of TET diagnosis (5-year OS: 76%).

The cause of death was confirmed in 57 of 65 cases. Six of the eight patients with unconfirmed cause of death had complete tumor resection and no progression, suggesting that death was not TET-related.

In patients with thymoma deaths were most frequently caused by other cancers (31%), while TET-specific death (90%) was predominant in patients with TC (Table 2, Supplementary Table 3). All deaths from other malignancies, treatment complications and autoimmune diseases were confined to patients with thymoma. Two deaths attributed to autoimmune complications were caused by myasthenic crisis and immunodeficiency-related infection, respectively.

**Table 2:** Cause of death among danish TET patients during the follow-up period with the contribution of the separate causes presented as the proportion of the total deaths during the follow-up period.

Cause of death	Thymoma (*N* = 45)	TC (*N* = 20)	Total (*N* = 65)
Autoimmune disease*	2 (4.4%)	0 (0%)	2 (3.1%)
**Cardiovascular disease**	4 (8.9%)	1 (5.0%)	5 (7.7%)
**Neurodegenerative disease**	1 (2.2%)	0 (0%)	1 (1.5%)
**Other cancer**	14 (31.1%)	0 (0%)	14 (21.5%)
**Pneumonia**	5 (11.1%)	1 (5.0%)	6 (9.2%)
**Tet**	9 (20.0%)	18 (90.0%)	27 (41.5%)
**Treatment complication**	2 (4.4%)	0 (0%)	2 (3.1%)
Unknown**	8 (17.8%)	0 (0%)	8 (12.3%)

For correlation of TET histology and TNM with survival and death, see Supplementary Table 2.

* Death caused by autoimmune diseases; one case of myasthenic crisis and one cases of severe infections in a patient with Goods Syndrome.

** Of the eight patients with an unknown cause of death, only two had active TET-disease at the time of death. TET: thymic epithelial tumours; TC: thymic carcinoma; TNM: Tumor, Node, Metastasis.

### Overall survival

**Figure 1:**
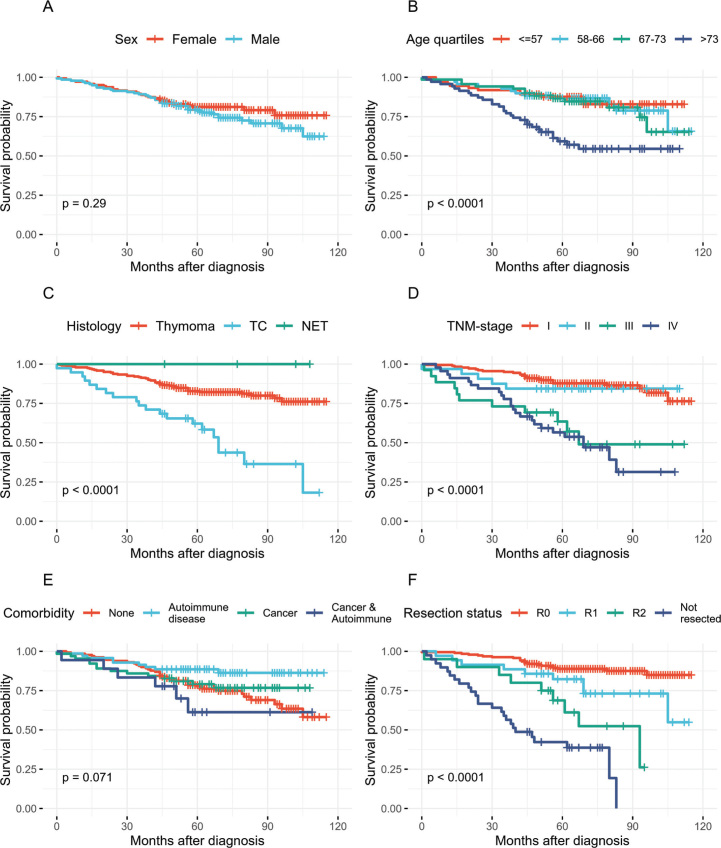
Kaplan–Meier curves for overall survival of Danish TET patients. (A–F) Overall survival stratified by sex, age quartiles, tumour histology, TNM-stage, pre-existing comorbidities and resection status. TET: thymic epithelial tumours; TC: thymic carcinoma; NET: neuroendocrine tumor; TNM: Tumor, Node, Metastasis.

OS did not differ notably between sexes (Figure 1A). In contrast, patients above 73 years of age diagnosis exhibited markedly reduced OS (Figure 1B).

Across histological subtypes, the lowest OS was observed in patients with TC (47%), compared with those diagnosed with thymoma (81.3%) or NETs (100%) (Figure 1C, Supplementary Table 3). The OS of patients with disease in TNM-stage I (86.0%) and II (84.0%) was notably higher than patients with disease in stage III (58%) and IV (47%) (Figure 1D). OS was slightly reduced in patients with pre-existing cancers (72%) and marginally improved in patients with pre-existing autoimmune diseases (83%), compared to that of the total cohort (77%) (Figure 1E).

Patients with R0 resected tumors had a notably higher OS (88%) than patients with R1 resections (74%), with the difference in OS approaching statistical significance (*p* = 0.053, χ^2^-test) (Figure 1F). In contrast, patients with macroscopically incomplete resections (R2) or no surgical intervention had markedly lower OS rates of 55 and 36%, respectively, highlighting the prognostic impact of complete resection.

### Prognostic factors of survival

In the multivariate analysis, only increasing age and advanced TNM stage (III or IV) at TET diagnosis remained significant prognostic factors for OS. Both factors were associated with increased mortality risk (Figure 1B & 1D, Table 3: age *p* = 0.01; TNM-stage III *p* = 0.02; TNM-stage IV < 0.001).

**Table 3:** Multivariate survival analysis performed using the Cox proportional hazards model.

Covariate	Cox proportional hazards model output		
	HR	95% CI	*P*
Age	1.04	1.01–1.06	0.01
Male sex	1.31	0.78–2.22	0.31
**Histology**			
A	1.00	Ref.	NA
AB	1.48	0.48–4.59	0.50
B1	1.13	0.29–4.39	0.86
B2	1.41	0.43–4.56	0.57
B3	1.38	0.33–5.69	0.66
NOS	3.02	0.73–12.46	0.13
Other thymoma	1.17	0.21–6.59	0.86
NET	0.00	0.00–Inf	1.00
TC	2.60	0.83–8.18	0.10
**TNM-stage**			
I	1.00	Ref.	NA
II	1.02	0.38–2.72	0.97
III	2.55	1.13–5.74	0.02
IV	4.27	2.13–8.56	0.00
**Comorbidities**			
Pre-existing cancer*	1.21	0.69–2.12	0.51
Pre-existing autoimmune disease**	1.16	0.60–2.22	0.66

* Patients with an established diagnosis of another primary cancer at the time of TET diagnosis.

** Patients with an existing autoimmune disease at the time of TET diagnosis. TET: thymic epithelial tumours; TC: thymic carcinoma; NET: neuroendocrine tumor; NOS: Not otherwise specified; TNM: Tumor, Node, Metastasis.

### Cancer specific survival

**Figure 2:**
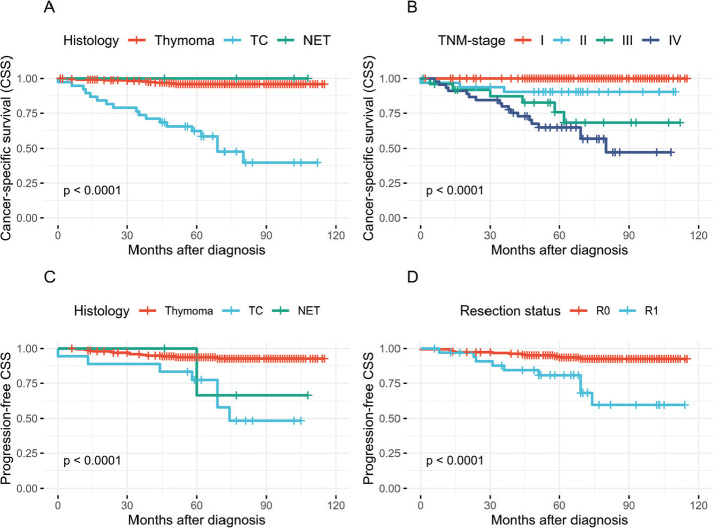
Kaplan–Meier curves for cancer-specific survival (CSS) and progression-free CSS of Danish TET patients. (A–B) Cancer specific survival by tumor histology and TNM-stage at diagnosis. (C–D) Progression-free CSS by tumor histology and resection status. Events were defined as death caused by TET or radiographically verified disease progression among 223 patients with R0 or R1 resected tumors. TET: thymic epithelial tumours; TC: thymic carcinoma; NET: neuroendocrine tumor; TNM: Tumor, Node, Metastasis.

The 5-year CSS was 63.2% in patients with TC and 96.3% patients with thymoma (Figure 2A). Patients with advanced TMN-stage had a poorer 5-year CSS (III: 80.8%, IV: 66.7%) than patients with early-stage disease (I: 100%, II: 90.6%) (Figure 2B).

### Relapse and PFS

TET relapse after R0 or R1 resection occurred in all histological tumor types and was radiographically verified in 13 of 201 (6.5%) patients with thymoma, 2 of 18 (11.1%) patients with TC and 1 of 4 (25%) patients with NET (Supplementary Table 2). The 5-year PFS was 94% for patients with thymoma and 77% for patients with TC (Figure 2C). In thymoma patients with R0 or R1 resection the 5-year PFS was 96.5 and 79.3%, respectively (Figure 2D).

## Discussion and conclusion

This population-based study presents survival outcomes and relapse in a nationwide cohort of Danish TET patients, offering an evaluation of prognostic factors in our patients.

This study benefits from a robust methodological framework, including a population-based design and centralization of care at one institution, ensuring standardized clinical and pathological assessments with minimal interobserver variability and near complete data. The comprehensive data availability of this study was enabled by the distinct layout of the Danish National Healthcare Registry, permitting linkage of patient files and collection of diagnostic and clinical data across all databases.

Although based on comprehensive data from a complete national cohort, the relatively small study population and the short follow-up limit our findings of prognostic markers. These limitations are expected to impact under-represented subgroups such as patients with NET, specific thymoma subtypes, and patients with disease progression after R0 or R1 resection, thus accentuating the importance of interpreting our results in the context of larger studies with longer time to follow-up and enhanced statistical power.

We report higher 5-year OS (76%) of Danish TET patients compared to the 5-year survival rates (69–70%) of historical European population-based cohorts [11, 19]. This higher OS of TET-patients is also seen in the Swedish (1958–2004) and South Korean (1999–2017) studies documenting changes in TET mortality over time [1, 11]. South Korean patients diagnosed with TET between 2013 and 2017 achieved a 5-year net survival of 91% for thymoma and 48% for TC [1], aligning closely with the 5-year CSS of the Danish cohort (Thymoma: 96%, TC: 53%), thereby supporting a trend over time towards improved survival outcomes of TET-patients across cohorts.

This trend may be explained by more patients diagnosed with low-stage disease, possibly driven by advancements in diagnostic procedures and the more frequent availability of CT-scans [20] or by improvements in treatment strategies and supportive care.

TET-specific mortality was accounted for the death within 5 years of diagnosis of 4.2 and 36.8% of Danish patients with thymoma and TC, respectively. This aligns with the findings of Shin et al. and Tseng et al. reporting the highest TET-specific mortality in patients with TC [1, 9]. Consistent with existing literature, all deaths from other cancers in our cohort were seen among patients with thymoma, supporting reports that this risk is more pronounced in thymoma compared to other TET types [1, 10]. Only two deaths were caused by autoimmune diseases, thus less than the 3.5–7% of autoimmunity-related deaths among patients with thymoma reported in previous studies [9, 21]. This discrepancy could be attributed to the shorter follow-up time of our cohort or recent advancements in treatment of autoimmune diseases, such as MG.

Increasing age and advanced TNM-stage (III or IV) were the only independent prognostic factors of OS in the multivariate analysis. This mirrors the results from a larger study of the TET patients in the American SEER database (*n* = 1224) [22], which also identified multiple other prognostic factors including sex, tumor histology, tumor size, surgery, and radiation therapy [22]. Other studies of American and South Korean TET patients found only age and tumor size to significantly influence OS [23, 24].

None of the studies found tumor histology to significantly impact OS of TET patients [22–24], despite TC being considered more malignant than thymoma [3]. This could be explained by the strong correlation between TC and advanced TNM-stage, possibly diminishing the effect of tumor histology in the multivariate analysis.

In line with existing literature, a higher OS was observed in patients with existing autoimmune diseases [9, 25]. The association did not persist in the multivariate analysis, likely due to the correlation between pre-existing autoimmunity and diagnosis of early-stage disease along with the predominance of thymomas.

Contrasting the findings of previous studies [10], pre-existing cancer was not and independent negative predictor of OS of Danish TET patients. This could reflect short follow-up, differences in tumor stage and histology distribution, or more frequent incidental detection of early-stage TETs in Danish patients regularly scanned for other cancers.

The discrepancy of the prognostic markers reported in the Danish cohort and in other populations might be attributed to our relatively short period of follow-up, small cohort size, demographic differences and discrepancies in clinical management of TETs across institutions. This highlights the need to analyze representative cohorts and interpret the results alongside larger studies to account for statistical limitation and demographic differences.

During the period of follow-up, 5% of the patients with R0 resections had radiographically verified TET relapse with a mean time to relapse of 33 months, thus aligning with the relapse rate (5.8%) and time to relapse (23 months) observed in thymectomized Chinese TET patients (*n* = 907) [26]. In their study, Liu et al. report a steady relapse rate in patients with type A, AB, and B1 thymoma throughout a 10-year period of follow-up [26], thereby accentuating the need for long-term follow-up of patients with thymoma.

The prevalence of recurrent cases across the histologic TET types reported in our study differs slightly from historical findings by others [4, 5, 9, 10]. These discrepancies are likely caused by the relatively few cases of relapse, possibly due to the short period of follow-up, and an under-representation of certain histological subtypes. Our reports of TET relapse should therefore be interpreted in the context of larger studies with enhanced statistical power.

In contrast to many other studies, we also investigated the rate of radiographically verified disease progression in R1 resected TET patients. Although cases of relapse after R1 resection were few in patients with thymoma (*n* = 6 of 29), the proportion of R1-resected patients presenting with disease progression during the time of follow-up (21%) was far greater than that of R0-resected patients (4%) (Figure 2B). This highlights the importance of complete tumor resection and accentuates the need to prioritize optimization of surgical strategies in TET management across all histologic types.

In conclusion, we report a high OS of Danish TET patients compared to previous population-based studies, possibly reflecting a broader increasing trend in survival. Still, the high prevalence of TET-related deaths in patients with TC and steady progression-rates after R0 and R1 resections in patients with thymoma, highlights the need for long-term surveillance of patients with TETs.

## Supplementary Material



## Data Availability

The data presented in this study are not publicly available. The data have been accessed through a study permit from the Capital Region of Denmark allowing the collection (R-20072336 + 21010159) and a permit from the Data Safety Authorities of the Capital Region of Denmark to store these retrospective data concerning the Danish TET patients (P-2020-1132).

## References

[1] Shin DW, Cho JH, Ha J, Jung KW. Trends in incidence and survival of patients with thymic epithelial tumor in a high-incidence Asian country: analysis of the Korean Central Cancer Registry 1999 to 2017. J Thorac Oncol. 2022;17(6):827–37. 10.1016/j.jtho.2022.02.001 35158083

[2] Østergaard T, Bjerke CVA, Santoni-Rugiu E, Jensen THL, Perell KA, Petersen RH, et al. Incidence, characteristics, and comorbidities of a complete unselected Danish cohort of patients with thymic epithelial tumors. Acta Oncol. 2025;64:41–6. 10.2340/1651-226X.2025.41295 PMC11758677 39813170

[3] Thoracic tumours. In: WHO Classification of Tumours Editorial Board (ed.). WHO classification of tumours. 5th ed. Lyon: IARC Publications; 2021. p. 319–399.

[4] Ruffini E, Detterbeck F, van raemdonck D, Rocco G, Thomas P, Weder W, et al. Tumours of the thymus: a cohort study of prognostic factors from the European Society of Thoracic Surgeons database. Eur J Cardio Thorac Surg. 2014;46(3):361–8. 10.1093/ejcts/ezt649 PMC4155438 24482389

[5] Weis CA, Yao X, Deng Y, Detterbeck FC, Marino M, Nicholson AG, et al. The impact of thymoma histotype on prognosis in a worldwide database. J Thorac Oncol. 2015;10(2):367–72. 10.1097/JTO.0000000000000393 25616178 PMC4318643

[6] Weksler B, Nason KS, MacKey D, Gallagher A, Pennathur A. Thymomas and extrathymic cancers. Ann Thorac Surg. 2012;93(3):884–8. 10.1016/j.athoracsur.2011.05.089 21962262

[7] Padda SK, Yao X, Antonicelli A, Riess JW, Shang Y, Shrager JB, et al. Paraneoplastic syndromes and thymic malignancies: an examination of the International Thymic Malignancy Interest Group Retrospective Database. J Thorac Oncol. 2018;13(3):436–46. 10.1016/j.jtho.2017.11.118 29191778 PMC5983900

[8] Benitez JC, Boucher ME, Dansin E, Kerjouan M, Bigay-Game I, Pichon E, et al. 53P Studying autoimmune diseases with thymic epithelial tumors (TET): real-world insight from RYTHMIC. Ann Oncol. 2020;31:S1437. 10.1016/j.annonc.2020.10.540

[9] Tseng YL, Chang JM, Lai WW, Chang KC, Lee SC, Lin SH, et al. Behind and beyond the masaoka staging: a 25-year follow-up study of tumor recurrence in completely resected thymic epithelial tumors in a single institution. Medicine. 2015;94(52):e2278. 10.1097/MD.0000000000002278 26717364 PMC5291605

[10] Hamaji M, Sozu T, MacHida R, Watanabe SI, Yoshida K, Toyooka S, et al. Mortality from extrathymic malignancy after thymic tumour resections: incidences and risk factors. Interact Cardiovasc Thorac Surg. 2019;29(5):729–36. 10.1093/icvts/ivz177 31326986

[11] Gadalla SM, Rajan A, Pfeiffer R, Kristinsson SY, Björkholm M, Landgren O, et al. A population-based assessment of mortality and morbidity patterns among patients with thymoma. Int J Cancer. 2011;128(11):2688–94. 10.1002/ijc.25583 20669226 PMC2992797

[12] Owe JF, Cvancarova M, Romi F, Gilhus NE. Extrathymic malignancies in thymoma patients with and without myasthenia gravis. J Neurol Sci. 2010;290(1–2):66–9. 10.1016/j.jns.2009.11.006 20034637

[13] Girard N, Ruffini E, Marx A, Faivre-Finn C, Peters S. Thymic epithelial tumours: ESMO clinical practice guidelines for diagnosis, treatment and follow-up. Ann Oncol. 2015;26:v40–55. 10.1093/annonc/mdv277 26314779

[14] Petersen PM, Kalhauge A, Brandt B, Santoni-Rugiu E, Daugaard G, Ravn J, et al. Behandling af tymom og thymuskarcinom. Ugeskrift Læger. 2020;182:2–6. 31928621

[15] Erichsen R, Lash TL, Hamilton-Dutoit SJ, Bjerregaard B, Vyberg M, Pedersen L. Clinical epidemiology existing data sources for clinical epidemiology: the Danish national Pathology registry and Data Bank. Clin Epidemiol. 2010;2:51–6. 10.2147/clep.s9908 20865103 PMC2943174

[16] UICC. TNM classification of malignant tumours. 8th ed. Brierley JD, editor. Geneva: UICC; 2016.

[17] The R Foundation for Statistical Computing. R statistical software [Internet]. Vienna: R Foundation for Statistical Computing; 2024 [cited 2024 Sep 3]. Available from: https://www.R-project.org/

[18] RStudio Team. RStudio [Internet]. Boston (MA): Posit Software, PBC; 2024. Available from: https://posit.co/

[19] de Jong WK, Blaauwgeers JLG, Schaapveld M, Timens W, Klinkenberg TJ, Groen HJM. Thymic epithelial tumours: a population-based study of the incidence, diagnostic procedures and therapy. Eur J Cancer. 2008;44(1):123–30. 10.1016/j.ejca.2007.11.004 18068351

[20] Borg M, Hilberg O, Andersen MB, Weinreich UM, Rasmussen TR. Increased use of computed tomography in Denmark: stage shift toward early stage lung cancer through incidental findings. Acta Oncol (Madr). 2022;61(10):1256–62. 10.1080/0284186X.2022.2135134 36264585

[21] Bernard C, Frih H, Pasquet F, Kerever S, Jamilloux Y, Tronc F, et al. Thymoma associated with autoimmune diseases: 85 cases and literature review. Autoimmun Rev. 2016;15(1):82–92. 10.1016/j.autrev.2015.09.005 26408958

[22] Li Y, Jiang A, Zhao Y, Shi C, Ma Y, Fu X, et al. A novel risk classifier for predicting the overall survival of patients with thymic epithelial tumors based on the eighth edition of the TNM staging system: a population-based study. Front Endocrinol (Lausanne). 2022;13:1050364. 10.3389/fendo.2022.1050364 36561557 PMC9763871

[23] Liu M, Wang C, Gao L, Lv C, Fu X. Clinical significance of age at diagnosis among patients with thymic epithelial tumors: a population-based study. Aging. 2020;12(6):4815–21. 10.18632/aging.102897 32224505 PMC7138550

[24] Yun JK, Kim HR, Kim DK, Shim YM, Kim YT, Chung KY, et al. Tumor size as a prognostic factor in limited-stage thymic epithelial tumors: a multicenter analysis. J Thorac Cardiovasc Surg. 2021;162(1):309–17.e9. 10.1016/j.jtcvs.2020.05.048 32736865

[25] Margaritora S, Cesario A, Cusumano G, Meacci E, D’Angelillo R, Bonassi S, et al. Thirty-five-year follow-up analysis of clinical and pathologic outcomes of thymoma surgery. Ann Thorac Surg. 2010; 89(1):245–52. 10.1016/j.athoracsur.2009.08.074 20103246

[26] Liu H, Gu ZT, Qiu B, Detterbeck FC, Roden AC, Ruffini E, et al. A recurrence predictive model for thymic tumors and its implication for postoperative management: a Chinese Alliance for Research in Thymomas Database Study. J Thorac Oncol. 2020;15(3):448–56. 10.1016/j.jtho.2019.10.018 31726106

