# Supplementary material for: Survival and relapse of Danish patients with thymic epithelial tumors
Source: Acta Oncol. 2026 May 21;65:45407. doi: 10.2340/1651-226X.2026.45407 (PMC13200513; doi:10.2340/1651-226X.2026.45407)
Supplement: Supplementary file 1 [file AO-65-45407-s1.pdf]

Supplementary material has been published as submitted. It has not been copyedited, or typeset by Acta Oncologica

Supplementary Material

TETs of combined histologic subtype	PATIENTS
**MIXED TC/THYMOMA***	
**50% B3, 50% TC**	1
**MIXED THYMOMAS****	
**90% B1, 10% B2**	2
**85% B1, 15% B2**	1
**80% B1, 20% B2**	1
**70% B1, 30% B2**	1
**60% B1, 40% B2**	1
**50% B1, 30% B2, 20% B3**	1
**90% B2, 10% B1**	1
**90% B2, 10% B3**	1
**80% B2, 20% B3**	2
**80% B2, 20% B3**	1
**70% B2, 30% B1**	1
**70% B2, 30% B3**	4
**70% B2, 20% B1, 10% B3**	1
**60% B2, 40% B3**	2
**50% B1, 50% B2**	3
**85% B3, 15% B2**	1
**80% B3, 20% B2**	1
**70% B3, 30% B2**	2
**60% B3, 40% B2**	2
**90% MICRONODULAR, 10% A**	1
**50% A, 50% MICRONODULAR**	1

Supplementary table 1: *Volume distribution of TETs of mixed histological subtype (2).*
** Tumors with both TC and thymoma components were clinically considered as TCs due to their more aggressive nature.*
*** Thymomas of mixed subtypes were grouped according to the volume contribution of each subtype.*

Supplementary Table 2: Survival, relapse, and comorbidities of Danish TET patients grouped by tumor histology.

	A (N=22)	AB (N=76)	B1 (N=44)	B2 (N=61)	B3 (N=14)	NOS* (N=10)	Other** thymoma (N=13)	NET (N=4)	TC (N=38)	Overall (N=282)
**Survival**										
**Alive**	18 (81.8%)	62 (81.6%)	39 (88.6%)	50 (82.0%)	10 (71.4%)	5 (50.0%)	11 (84.6%)	4 (100%)	18 (47.4%)	217 (77.0%)
**Dead**	4 (18.2%)	14 (18.4%)	5 (11.4%)	11 (18.0%)	4 (28.6%)	5 (50.0%)	2 (15.4%)	0 (0%)	20 (52.6%)	65 (23.0%)
**Relapse**										
**Radiologic progression**	0 (0%)	2 (2.6%)	4 (9.1%)	7 (11.5%)	0 (0%)	0 (0%)	0 (0%)	1 (25.0%)	2 (5.3%)	16 (5.7%)
**No radiologic progression**	22 (100%)	71 (93.4%)	36 (81.8%)	49 (80.3%)	8 (57.1%)	10 (100%)	13 (100%)	3 (75.0%)	34 (89.5%)	246 (87.2%)
**R2 or no resection**	0 (0%)	3 (3.9%)	4 (9.1%)	5 (8.2%)	6 (42.9%)	0 (0%)	0 (0%)	0 (0%)	2 (5.3%)	20 (7.1%)
**Existing cancer**										
**No**	9 (40.9%)	41 (53.9%)	30 (68.2%)	44 (72.1%)	12 (85.7%)	4 (40.0%)	7 (53.8%)	2 (50.0%)	28 (73.7%)	177 (62.8%)
**Yes**	13 (59.1%)	35 (46.1%)	14 (31.8%)	17 (27.9%)	2 (14.3%)	6 (60.0%)	6 (46.2%)	2 (50.0%)	10 (26.3%)	105 (37.2%)
**Existing autoimmune disease**										
**No**	14 (63.6%)	55 (72.4%)	27 (61.4%)	36 (59.0%)	5 (35.7%)	8 (80.0%)	11 (84.6%)	4 (100%)	34 (89.5%)	194 (68.8%)
**Yes**	8 (36.4%)	21 (27.6%)	17 (38.6%)	25 (41.0%)	9 (64.3%)	2 (20.0%)	2 (15.4%)	0 (0%)	4 (10.5%)	88 (31.2%)

***Supplementary table 2:** The survival, relapse-rate and prevalence of autoimmune diseases and other primary cancers are shown for patients with each histological subtype.*
**Not otherwise specified thymoma (NOS): includes patients without subtype classification because of insufficient tissue from biopsy.*
***Other thymoma: includes patients diagnosed with micronodular and metaplastic thymomas.*

Supplementary table 3: Cause of death and disease characteristics

CAUSE OF DEATH	TUMOR HISTOLOGY	TNM-STAGE	PATIENTS	PROPORTION*
**Cardiovascular disease**	AB	I	2	(3.3%)
	AB	IV	1	(33.3%)
	B2	I	1	(2.6%)
	TC	I	1	(16.7%)
**Complication to autoimmune disease**	AB	II	1	(10.0%)
	B2	II	1	(14.3%)
**Neurodegenerative disease**	AB	I	1	(1.6%)
**Other cancers**	AB	I	4	(6.6%)
	B1	I	5	(14.3%)
	B2	I	2	(5.3%)
	B3	IV	1	(20.0%)
	Micronodular	I	1	(10.0%)
	NOS**	III	1	(25.0%)
**Pneumonia**	A	IV	1	(50.0%)
	AB	I	1	(1.6%)
	AB	IV	1	(33.3%)
	B2	III	1	(12.5%)
	B3	IV	1	(20.0%)
	TC	III	1	(14.3%)
**TET**	AB	IV	1	(33.3%)
	B2	IV	5	(62.5%)
	NOS	II	1	(50.0%)
	NOS	III	2	(50.0%)
	TC	II	2	(40.0%)
	TC	III	4	(57.1%)
	TC	IV	12	(60.0%)
**Unknown**	A	I	3	(16.7%)
	AB	I	2	(3.3%)
	B3	IV	1	(20.0%)
	Micronodular	I	1	(10.0%)
	NOS	I	1	(50.0%)

Supplementary table 3: *association between cause of death, tumor histology, and official TNM stage.*
** The number of patients who died from specific causes is shown as a proportion of the patients with the same stage and histological type.*
*** Not otherwise specified thymoma (NOS): includes patients without subtype classification due to insufficient tissue from biopsy.*
